# Falls prevention at GP practices: a description of daily practice

**DOI:** 10.1186/s12875-021-01540-7

**Published:** 2021-09-21

**Authors:** Wytske M. A. Meekes, Chantal J. Leemrijse, Yvette M. Weesie, Ien A. M. van de Goor, Gé A. Donker, Joke C. Korevaar

**Affiliations:** 1 Tranzo, Tilburg School of Social and Behavioral Sciences, Tilburg University, Postbus 90153, 5000 LE Tilburg, Netherlands; 2 NIVEL, Otterstraat 118-124, 3513 CR Utrecht, Netherlands

**Keywords:** Falls prevention, Primary care, Frail older people

## Abstract

**Background:**

General practitioners (GPs) can be considered the designated professionals to identify high fall risk and to guide older people to fall preventive care. Currently it is not exactly known how GPs treat this risk. This study aims to investigate GPs’ daily practice regarding fall preventive care for frail older patients.

**Methods:**

Sixty-five GPs from 32 Dutch practices participated in this study for a period of 12 months. When a GP entered specific International Classification of Primary Care-codes related to frailty and/or high fall risk in their Electronic Health Records, a pop-up appeared asking “Is this patient frail?”. If the GP confirmed this, the GP completed a short questionnaire about patient’s fall history and fear of falling (FOF), and the fall preventive care provided.

**Results:**

The GPs completed questionnaires regarding 1394 frail older patients aged ≥75. Of 20% of these patients, the GPs did not know whether they had experienced a fall or not. The GPs did not know whether a FOF existed in even more patients (29%). Of the patients with a fall history and/or a FOF (*N* = 726), 37% (*N* = 271) received fall preventive care. Two main reasons for not offering fall preventive care to these patients were: I) the patient finds treatment too intensive or too much of a hassle (37%), and II) the GP identified a high fall risk but the patient did not acknowledge this (14%). When patients were treated for high fall risk, the GP and the physiotherapist were the most frequently involved health care providers. The involved health care providers most often treated mobility limitations, cardiovascular risk factors, and FOF.

**Conclusions:**

The results from this study show that GPs were frequently not aware of their frail patient’s fall history and/or FOF and that the majority of the frail older patients with a fall history and/or FOF did not receive fall preventive care. Developing systematic screening strategies for the primary care setting enhancing the identification of high fall risk and the provision of fall preventive care may improve patients’ quality of life and reduce health care costs.

**Supplementary Information:**

The online version contains supplementary material available at 10.1186/s12875-021-01540-7.

## Background

Falls are a major health threat for older people [1]. On average, 30% of people aged ≥65 and 42% of the people aged ≥75 fall at least once a year [2–5]. The consequences of a fall can vary, from a bruise to a brain injury [6] and may have a long-lasting negative impact on an older person’s independence and quality of life [1, 3]. A fall may even cause death, resulting worldwide in approximately 646,000 deaths annually [1]. In 22 Western European countries, the death rate varied from 27 to 153 per 100,000 people aged ≥70 in 2017 [7]. These numbers will increase further as society is aging. Additionally, falls may lead to considerable medical costs [1, 8].

Frail older people are an important group to focus on in regards to falls prevention, as frailty appears to be an important risk factor for falls [9]. For example, Bandeen-Roche et al. (2015) found that the percentages of people who experienced a fall in the previous year or had a fear of falling (FOF), are three to four times higher among frail older people compared to robust older people [10]. A FOF can be defined as an ongoing concern about falling that ultimately limits the individual’s performance of daily activities [11]. Furthermore, many other fall risk factors can also be associated with frailty, such as reduced balance and strength, use of sleep medication, and dizziness [12, 13].

There are several evidence-based falls prevention interventions available to reduce fall risk by tackling the risk factors [14–17]. Falls prevention interventions differ in approach, target group, intensity, and type of treatment. Overall, multifactorial programs with evidence-based interventions are recommended to reduce fall risk [18, 19]. It is important that qualified health care professionals guide older people to participate in these falls prevention interventions, because older people often do not realize they have a high fall risk and are unfamiliar with available interventions to reduce this risk [20, 21].

In the Netherlands, general practitioners (GPs) act as gatekeepers to secondary health care and they are often the first point of contact when independently living, frail older people encounter health issues [22, 23]. Furthermore, GPs have insight in a patient’s medical history, home setting, and social network, making them the designated health care professionals for identifying high fall risk and providing falls prevention.

At the moment, it is not exactly known which type of care GPs offer to frail older people to reduce their high fall risk. It is also unclear what proportion of the frail older patients receives this care or why they do not receive fall preventive care. Therefore, this study aims to investigate GPs’ daily practice regarding fall preventive care for frail older patients. The following research questions will be answered:

1. Which and how many frail older patients receive fall preventive care and what are the reasons for not providing falls prevention to frail older patients with a fall history and/or fear of falling?
2. Which fall risk factors are treated among frail older patients and which health care professionals are involved in the provision of fall preventive care?

## Methods

### Study design

In this prospective cohort study, a network of 65 GPs (43.7 fulltime-equivalent) from 32 sentinel practices participating in Nivel Primary Care Database, participated for a period of 12 months in 2018. Since 1970, GP practices were recruited and selected (after application) to participate in the Nivel Primary Care Database based on region and population density of their practice in order to ensure national representation of patients. The participating GPs assess and deliver data regarding certain illnesses, events and procedures in GP practices. The majority of the participating GP practices are located in urbanized rural municipalities (15,6% rural [< 500 patients/km2], 62,5% urbanized rural [500–2500 patients/km2], 21,9% urban [> 2500 patients/km2]). Slightly less participating GPs have a partnership with ≥2 GPs (58.2%) compared to all Dutch GP practices (65.9%). In total, the GP practices have 112.171 patients (μ = 3505, min = 1450, max = 9800), 0.7% of the Dutch population.

For this study, the GPs completed a questionnaire concerning fall preventive care for their frail older patients. Patients from these GPs are representative of the entire Dutch population regarding age, sex, regional distribution, and degree of urbanization and cover in our study 0.7% of the Dutch population. More information about this GP network can be found in Donker (2019) and Schweikardt, Verheij, Donker, & Coppieters (2016) [24, 25].

### Data collection

The participating GPs report all their patient data in their Electronic Health Records (EHR) by using among others International Classification of Primary Care (ICPC)-codes version 1 [26]. The Dutch GP Association, ‘NHG’, provides guidelines how to adequately register data in the EHR [27]. Inclusion of patients in this study was based on specific ICPC-codes entered in the EHR.

The research team, together with two GPs selected ICPC-codes that may be related to frailty and/or high fall risk (e.g. fractures, visual impairment, balance problems) based on previous literature [28–30], see Additional file 1. When the participating GPs entered one of the selected ICPC-codes into the EHR concerning a patient aged ≥75, a pop-up appeared with the question “Is this patient frail?”. If the GP answered ‘Yes’, based on clinical judgement or a frailty screening instrument, this GP received a questionnaire with 6 items regarding patient’s fall history and FOF, and the fall preventive care provided (see Additional file 2). The GPs could complete the questionnaire directly, or later at a more convenient time.

### Questionnaire

In this study, a 6-item multiple choice questionnaire was developed based on previous literature (see Additional file 2). The questionnaire had to be short and should take less than 5 minutes to complete, to fit it into the routine daily practices of GPs.

Question 1, regarding frailty, was based on literature that showed that healthcare professionals can assess frailty with instruments such as the Tilburg Frailty Indicator [31] and the Groninger Frailty Indicator [32], but also on their clinical judgement [33]. Question 2 was based on several studies describing that a patient’s fall history or FOF can indicate future falls [34–40]. The remaining questions, concerning the inventory of underlying causes of the high fall risk and the health care providers involved in the provision of falls prevention interventions, were based on the Dutch guidelines regarding falls prevention and previous literature [15, 28, 30].

### Analysis

The data were analysed, using descriptive statistics in STATA [41]. To identify significant differences in patients’ sex, the χ^2^ – test was used and for significant differences in age the two-sample t-test was used. In this article, only the significant differences with a *p*-value below 0.05 are reported.

## Results

### Characteristics

In this study, 1512 eligible patients were identified as frail. The GPs completed questionnaires regarding 1394 patients. Of 118 patients, the questionnaire was missing. There were no significant differences in age and sex distribution between the frail older patients with (*N* = 1394) and without the questionnaires (*N* = 118). The GPs diagnosed 343 (24,6%) of the 1394 patients as being frail based on instruments that screen or diagnose frailty. The other remaining 1051 frail patients were diagnosed based on the GPs’ clinical judgement.

Table 1 describes the characteristics of all 1,394 frail older patients and the subgroup of 726 (52%) frail older patients who experienced a fall in the previous year and/or who had a FOF.

**Table 1:** Characteristics all frail patients and the frail patients with fall history and/or FOF

	All frail patients			Frail patients with fall history and/or FOF		
	Total	Male	Female	Total	Male	Female
	1394	505	889	726	235	491
Mean age	84.5	83.8	84.9	85.0	84.7	85.1
(SD, range)	(5.81; 75–102)	(5.65; 75–100)	(5.87; 75–102)	(5.82 75–101)	(5.88 75–100)	(5.79 75–101)
ICPC code related to						
**A.** General						
Feeling ill/Overall decline/Frailty	24.4%	26.5%	23.2%	16.9%	17.9%	16.5%
Fainting/Syncope	3.5%	4.6%	2.9%	4.0%	6.0%	3.1%
Trauma/ Injury NOS/ Falls	7.5%	6.1%	8.2%	12.1%	10.2%	13.0%
Elderly care/Preventive operations	6.3%	5.3%	6.9%	5.4%	4.3%	5.9%
**F.** Eye	1.4%	1.2%	1.5%	1.8%	0.9%	2.2%
**H.** Ear	2.4%	2.6%	2.4%	2.6%	2.6%	2.6%
**K.** Circulatory	26.5%	31.3%	23.7%	23.7%	28.9%	21.2%
**L.** Muscoloskeletal	25.5%	17.0%	30.4%	28.7%	21.3%	32.2%
**N.** Neurological	10.5%	12.1%	9.7%	13.6%	16.2%	12.4%

**Fig. 1:**
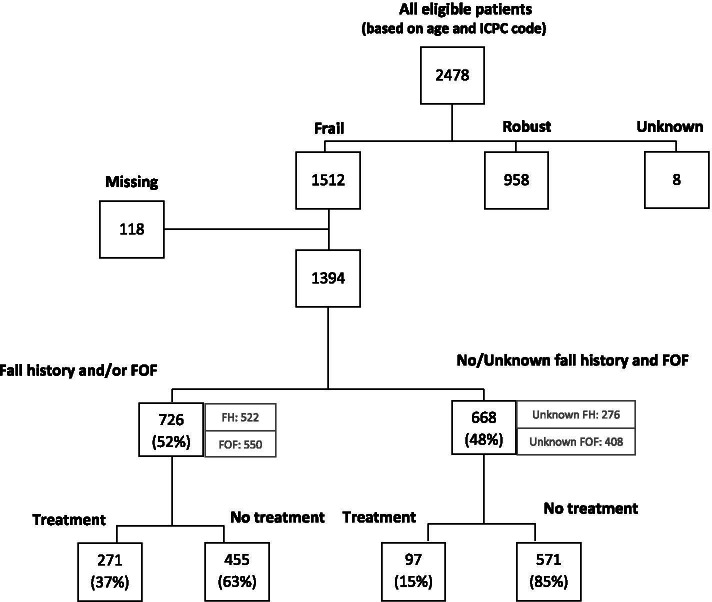
Fall preventive care offered to frail older patients

The GPs reported that in total 522 frail older patients (37%) experienced a fall in the previous year and 550 frail older patients (39%) had a FOF, see Fig. 1 (for a more precise flowchart see Additional file 3). The GPs did not know of 276 frail patients (20%) whether they experienced a fall in the previous year, and of 408 patients (29%) whether they had a FOF.

Sixty-six percent (346/522) of the patients that experienced a fall in the previous year also had a FOF, which is significantly more than the patients who did not experience a fall (27%) (*p* < 0.01). In addition, more females than males had a FOF (*p* < 0.01).

### Fall preventive care

According to the GPs, 726 frail older patients experienced a fall in the previous year and/or had a FOF. The GPs reported that 271 (37%) of these 726 patients, received fall preventive care. Two hundred six patients (39%) from the 522 patients with a fall in the previous year, and 215 patients (39%) from the 550 patients with a FOF, received fall preventive care. Fifteen percent of the patients without and/or an unknown fall history or FOF also received fall preventive care (see Fig. 1 and Additional file 3).

**Fig. 2:**
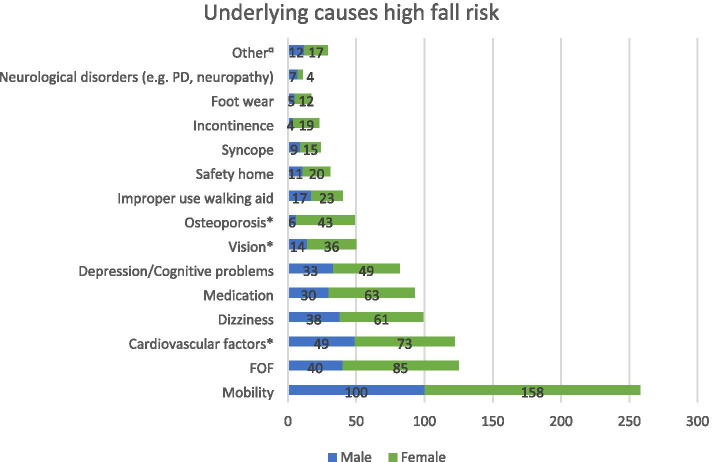
Underlying causes of high fall risk of all frail older patients receiving fall preventive care. *Significant differences in sex (*p* < 0.05). ^a^Other: alcohol abuse (8), revalidation after a fall or surgery (4), recent fracture (4), osteoarthritis (3), pain (3), renal failure (2), weakness due to cancer (2), COPD (1), sarcopenia (1), hearing problems (1), death (1)

The five most treated fall risk factors among patients receiving fall preventive care were according to the GPs I) limitations in mobility (*N* = 258), II) FOF (*N* = 125), III) cardiovascular risk factors (*N* = 122), IV) dizziness (*N* = 99), and V) medication (*N* = 93) (Fig. 2). More females were treated for osteoporosis (*p* < 0.01) and more males for alcohol abuse (*p* = 0.02). Patients treated for alcohol abuse were on average younger (*p* < 0.01, μ = 84.49, SD = 5.812) and patients receiving safety adjustments at home were on average older (*p* < 0.01, μ = 84.49, SD = 5.812).

**Fig. 3:**
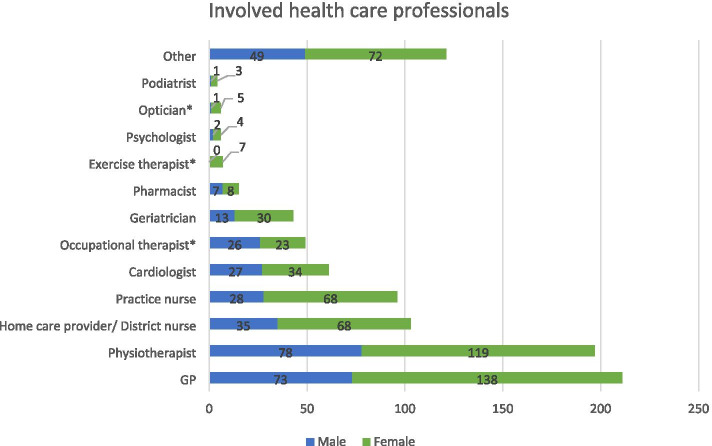
Health care professional involved in the provision of fall preventive care to all frail older patients. * Significant differences (*p* < 0.05). ^a^Other: neurologist (21), ophthalmologist (17), orthopedist (13), case-manager (11), internist (8), nursing home health care professionals (8), geriatrician/ geriatric specialist (4), family (4), GP-assistant (3), surgeon (3), oncologist (2), rheumatologist (2), rehabilitation doctor (2)

The five health care providers, who are most frequently involved in the provision of falls prevention, were I) the GP (*N* = 211), II) the physiotherapist (*N* = 197), III) the home care provider/district nurse (*N* = 103), IV) the practice nurse (*N* = 96), and V) the cardiologist (61) (Fig. 3). Significantly more females than males received care from occupational therapists (*p* = 0.013) and exercise therapists (*p* = 0.046).

Of patients who had experienced a fall in the previous year and/or had a FOF according to their GP, the majority, 455 (63%), did not receive fall preventive care. The main reasons for not offering fall preventive care to these patients were, according to the GPs, I) the patient finds treatment too intensive or too much of a hassle (37%, *N* = 170), II) the GP identified a high fall risk but the patient did not acknowledge this (14%, *N* = 64), and III) the patient did not have a high fall risk according to both the clinical judgement of the GP and the patient (13%, *N* = 51) (see Table 2).

**Table 2:** Reasons for not providing fall preventive care to frail older patients

Reason for not providing fall preventive care	All male patients	All female patients	Male patients with FH and/or FOF	Female patients with FH and/or FOF
1. No high fall risk	157	242	15	38
2. Both agree on high fall risk, but patient beliefs treatment is too intense/too much of a hassle	63	153	49	121
3. GP diagnoses high risk but patient does not acknowledge this	48	77	23	41
4. GP forgot/had no time to discuss fall prevention with the patient	10	37	4	25
5. GP explained that diagnosis of the high fall risk still ongoing	14	21	2	2
6. Patient was too sick/weak for treatment according to GP	14	19	8	10
7. Patient was in or going to a hospital/health care facility/rehabilitation center and therefore will receive care from other health care professionals	12	16	9	12
8. Patient has or had already received fall preventive care	9	17	7	14
9. Patient has other health issues with higher priority	6	14	4	5
10. Patient is barely/not mobile and therefore treatment is not necessary/possible	5	12	0	6
11. Patient has dementia	5	11	3	8
12. Treatment costs are too high	5	6	4	4
13. Other^a^	16	32	12	23
14. Unclear	15	22	5	15

Note. GPs could give more than one reason for not providing fall preventive care

^a^Remaining (reasons given regarding ≤10 patients for each category): according to the GP there is no improvement possible by providing fall preventive care, patient has already aids (e.g. walker, personal alarm), the patient passed away, the fall of the patient was a single event, the patient takes own measures to reduce fall risk, and 15 singular reasons

Other reasons for not providing fall preventive care can be divided in the following five categories: I) the patient is too weak, sick, cognitively impaired or not mobile, II) the patient is hospitalized, institutionalized or deceased, III) the patient already received fall preventive care or mobility aids, IV) the GP forgot to discuss falls prevention with the patient, other health priorities prevailed or the GP expects no effect from providing fall preventive care, and V) the patient takes own measures to reduce fall risk. Of 20 patients with a fall history and/or FOF, information about not offering fall preventive care was lacking.

## Discussion

Our study aimed to investigate GPs’ daily practice regarding the provision of falls prevention to frail older patients. The results show that the participating GPs reported a fall in the previous year and/or a FOF among half of the frail older patients (aged ≥75) who contacted the GP practices during the 12 months of this study. Thirty-seven percent of these patients with a fall history and/or a FOF received fall preventive care. The main reason for not providing fall preventive care to these patients was that the patients believed that the treatment is too intensive or too much of a hassle. Also, a high fall risk was not always acknowledged by patients and/or GPs, even though patients had experienced a fall in the previous year and/or had a FOF. When patients were treated for high fall risk, the GP and the physiotherapist were the most frequently involved health care providers. The health care providers most often treated mobility limitations, cardiovascular risk factors, and FOF.

The participating GPs reported that 37% (522 patients) of their frail older patients had experienced a fall in the previous year. It seems that in our study the GPs identified more frail older people with a fall history than in other GP practices [42–44]. According to Schoon (2013) and Stel et al. (2004), GPs are only aware of 20–30% of the falls of their patients because older patients most often do not inform their GPs about their falls [42, 44]. In the study of Stel et al. (2004), only 21% of the 204 participants aged > 65 visited the GP after their fall [44]. The differences with our study could be explained by environmental differences and population differences (e.g. age, sex, level of frailty) but also by our methods. In our study, GPs were actively asked about their patients’ fall history and the GPs may also have directly asked their patients if they had experienced a fall in the previous year even when the patient did not visit the GP regarding a fall.

Nevertheless, we expect that probably more than the 37% of the frail older people, found in our study, experienced a fall in the previous year. Bandeen-Roche et al. (2015) reported in their study that 55% of their 1138 frail older participants aged 65–89 experienced a fall in the previous year [10]. And Ensrud et al. (2007) described that 41% of their 1096 frail older female participants aged ≥69 (μ76.7, SD ± 4.9) experienced a fall in the previous year [45]. The percentages in our study are lower which might be explained by environmental differences, population differences (e.g. age, sex, level of frailty), methodological differences, and how frailty was assessed in these studies. Even though possibly not all frail older people with a high fall risk were identified by using our study design, the results imply that routinely asking patients about their fall history and FOF, may help to identify frail older people with a fall history and a possible high fall risk. The strategy used in our study, a pop-up in the registration system during consultation, might be implemented to support GPs to routinely ask these questions.

This study shows that the majority of the patients with a fall history and/or FOF do not receive fall preventive care. Campbell & Robertson (2006) describe that there is a common misconception about older people being too frail to participate in falls prevention interventions [46]. Also in our study, it appears that some reasons for not providing fall preventive care could be related to the patients’ frailty, such as patients’ poor health, comorbidities and their beliefs or experiences that interventions are too intensive. These results indicate that the current available interventions and treatments may not be suitable for some older people who are (severely) frail. They may also point to the advantage of offering interventions in an earlier phase when people are not (yet) or less frail [47]. Starting with falls prevention in an earlier phase may enhance healthier ageing and staying longer independent.

Another main reason reported by the GPs for not providing fall preventive care was lack of acknowledgement of high fall risk by patients even though the GP diagnosed it. The percentage of older patients not acknowledging their high fall risk will probably increase when targeting patients who are not yet or less frail. Helping patients to understand why they have a high or increased fall risk and what measures can be taken, are important to motivate patients to start falls prevention interventions. Previous literature shows that older people are often unfamiliar with the meaning of high fall risk and what measures can be taken to reduce this risk [20, 21]. In addition, possible stigma exists around falling among older people as it can be related to aging and loss of independence which again might negatively influence their motivation to participate in falls prevention interventions [48, 49]. Therefore, changing the focus from preventing falls to promoting a healthier life style and maintaining independence might stimulate older people to start and complete falls prevention interventions.

### Further research

The results from our study show that GPs did know of 80% of their patients if they had a fall history and of 71% if they had a FOF, which can indicate high fall risk. However, the majority (63%) of the patients with a fall history and/or FOF did not receive fall preventive care. Further research is recommended to investigate whether the number of patients that receive fall preventive care can be increased. According to Paul, a systematic approach for identifying risks and addressing them in a concerted manner is key to falls prevention [50]. Therefore, strategies for systematic fall risk screening and falls prevention provision in the primary care setting could be developed, implemented and evaluated to reduce falls among frail, independently living older people. Such strategies should take older people’s perspectives, needs, and wishes into account and focus on start and compliance of interventions [51, 52]. Also, the roles of the GP and other involved health care professionals should be further investigated in regards to systematic fall risk screening and falls prevention provision.

The GPs reported in this study several reasons why they did not provide falls prevention to patients with a fall history and/or FOF. Further research is required to gain in depth knowledge for not providing fall preventive care. This is relevant information to make the implementation strategies more successful. If more frail older people with high fall risk receive adequate fall preventive care, falls and FOF among patients can be reduced [17, 53]. In addition, their quality of life, and independence may be maintained or improved. Moreover, these health outcomes can result in a reduction in hospital admissions, rehabilitation treatments, and other related treatments, which finally could lead to a reduction in health care costs [54].

### Strengths and limitations

A limitation of our study is the data collection itself. The data collection can be seen as an intervention. GPs received a pop-up in their registration system asking about patient’s frailty, fall history and FOF when reporting specific ICPC-codes in their EHR. These questions will have made GPs more aware about frailty and falls prevention resulting in more active GPs concerning the provision of falls prevention. Therefore, the results from our study might be an overestimation of the actual proportion of patients with a fall history and/or FOF because normally GPs will not systematically ask these questions during daily practice. Nevertheless, the proportion of frail older people identified with a fall history and/or FOF appears to decrease when comparing the periods January until July and July until December (not significantly), indicating that the GPs’ initially increased awareness declines slightly over time.

There might also be some information bias in our study. When the GPs completed the questionnaire, they might have asked patients directly, assumed the answer or retrieved answers from the EHR. GPs assuming or patients recalling answers might have caused bias during data collection.

On the contrary, the method of data collection is also a strength of our study. The questionnaire was incorporated in the EHR, enhancing more complete data collection as the system helped reminding the GPs to complete the questionnaire.

Another strength of our study is related to registration performance of the GP practices. In general, the quality of registration varies among Dutch GPs [55]. Nonetheless, the participating GP practices were classified as practices with good registration performance because they have registered ICPC codes after at least 70% of their contacts with patients and at least 46 weeks of adequate contact registration during a period of 1 year.

The network of 32 GP practices (65 GPs, 43.7 fulltime-equivalent) throughout the Netherlands, used in this study, was also a strength. Patients in this network are representative of the entire Dutch population regarding age, sex, regional distribution, and degree of urbanization and cover 0.7% of the Dutch population [56].Therefore, our study gives a good representation of daily practice of Dutch GPs across the country in regards to the provision of falls prevention [24].

## Conclusion

In conclusion, this study shows that the GPs are frequently not aware of their patient’s fall history and/or FOF and that the majority of the frail older patients with a fall history and/or FOF did not receive fall preventive care. Main reasons for not providing fall preventive care are related to the patients’ beliefs that interventions are too intensive/too much of a hassle, and the lack of patients’ acknowledgement of their high fall risk. This situation could be improved by developing strategies for the primary care setting that aim to enhance systematic screening of high fall risk and provision of falls prevention among frail older people as well as among patients who are less frail. In addition, further research is required to gain more in-depth insights in why fall preventive care is not provided. With all this, falls prevention can be implemented more successfully in the primary care setting which helps to reduce falls among older patients. This may lead to a maintained or improved quality of life of frail older people and a reduction in health care costs.

## Supplementary Information

**Additional file 1.** Selected ICPC-codes related to frailty and/or high fall risk

**Additional file 2.** Questionnaire used to investigate GPs daily practice regarding falls prevention

**Additional file 3.** Flowchart fall preventive care offered to frail older patients

## Data Availability

According to the Governance of Nivel Primary Care Database, the Steering committee with representatives from national associations of general practitioners decide about the use of the data. Therefore, permission from this Steering committee is required for each request to do research with the data.

## Abbreviations

EHR: Electronic Health Records
FOF: Fear of Falling
GP: General Practitioner
ICPC: International Classification of Primary Care

## References

[1] World Health Organization: Falls: fact sheet, 2018. Accessed 25 Jun 2018.

[2] Deandrea S, Lucenteforte E, Bravi F, Foschi R, La Vecchia C, Negri E. Risk factors for falls in community-dwelling older people: “a systematic review and meta-analysis”. Epidemiology. 2010:658–68. 10.1097/EDE.0b013e3181e89905 20585256

[3] Gosney M, Harper A, Conroy S (2012). Oxford desk reference: geriatric medicine.

[4] Tinetti ME (2001). Where is the vision for fall prevention?. J Am Geriatr Soc.

[5] Tinetti ME, Speechley M (1989). Prevention of falls among the elderly. N Engl J Med.

[6] Terroso M, Rosa N, Marques AT, Simoes R (2014). Physical consequences of falls in the elderly: a literature review from 1995 to 2010. Eur Rev Aging Phys A.

[7] Haagsma JA, Olij BF, Majdan M, Van Beeck EF, Vos T, Castle CD, et al. Falls in older aged adults in 22 European countries: incidence, mortality and burden of disease from 1990 to 2017. Injury Prev. 2020. 10.1136/injuryprev-2019-043347 PMC7571349 32111726

[8] Van der Does H BA, Panneman M Privé-ongevallen bij ouderen, Cijfers valongevallen in de privésfeer 2018. In.: VeiligheidNL; 2019.

[9] Cheng MH, Chang SF (2017). Frailty as a risk factor for falls among community dwelling people: evidence from a meta-analysis. J Nurs Scholarsh.

[10] Bandeen-Roche K, Seplaki CL, Huang J, Buta B, Kalyani RR, Varadhan R, Xue Q-L, Walston JD, Kasper JD (2015). Frailty in older adults: a nationally representative profile in the United States. J Gerontol A.

[11] Tinetti ME, Powell L. Fear of falling and low self-efficacy: a cause of dependence in elderly persons. J Gerontol. 1993. 10.1093/geronj/48.special_issue.35 8409238

[12] Boelens C, Hekman EE, Verkerke GJ (2013). Risk factors for falls of older citizens. Technol Health Care.

[13] Pfortmueller C, Lindner G, Exadaktylos A (2014). Reducing fall risk in the elderly: risk factors and fall prevention, a systematic review. Minerva Med.

[14] Balzer K, Bremer M, Schramm S, Lühmann D, Raspe H. Falls prevention for the elderly. GMS Health Technol Assess. 2012;8. 10.3205/hta000099 PMC3334922 22536299

[15] Hopewell S, Adedire O, Copsey BJ, Boniface GJ, Sherrington C, Clemson L, et al. Multifactorial and multiple component interventions for preventing falls in older people living in the community. Cochrane Database Syst Rev. 2018;(7). 10.1002/14651858.CD012221.pub2 PMC6513234 30035305

[16] Sherrington C, Fairhall NJ, Wallbank GK, Tiedemann A, Michaleff ZA, Howard K, et al. Exercise for preventing falls in older people living in the community. Cochrane Database Syst Rev. 2019;(1). 10.1002/14651858.CD012424.pub2 PMC6360922 30703272

[17] Tricco AC, Thomas SM, Veroniki AA, Hamid JS, Cogo E, Strifler L, Khan PA, Robson R, Sibley KM, MacDonald H (2017). Comparisons of interventions for preventing falls in older adults: a systematic review and meta-analysis. JAMA.

[18] Grossman DC, Curry SJ, Owens DK, Barry MJ, Caughey AB, Davidson KW, Doubeni CA, Epling JW, Kemper AR, Krist AH (2018). Interventions to prevent falls in community-dwelling older adults: US preventive services task force recommendation statement. JAMA.

[19] Luk JK, Chan T, Chan DK (2015). Falls prevention in the elderly: translating evidence into practice. Hong Kong Med J.

[20] Shankar KN, Taylor D, Rizzo CT, Liu SW (2017). Exploring older adult ED fall patients’ understanding of their fall: a qualitative study. Geriatr Orthop Surg Rehabil.

[21] Yardley L, Donovan-Hall M, Francis K, Todd C (2006). Older people’s views of advice about falls prevention: a qualitative study. Health Educ Res.

[22] Boerma WGW (2003). Profiles of general practice in Europe: an international study of variation in the tasks of general practitioners.

[23] Schellevis FG, Westert GP, De Bakker DH (2005). The actual role of general practice in the Dutch health-care system. J Public Health.

[24] Donker: Nivel Zorgregistraties Eerste Lijn-Peilstations 2018. 2019.

[25] Schweikardt C, Verheij RA, Donker GA, Coppieters Y (2016). The historical development of the Dutch sentinel general practice network from a paper-based into a digital primary care monitoring system. J Public Health.

[26] Lamberts H, Wood M (2002). The birth of the international classification of primary care (ICPC) serendipity at the border of lac leman.

[27] Duineveld BKH, Van Werven H, Njoo KH (2019). NHG-Richtlijn, Adequate dossiervorming met hetelektronisch patiëntdossier (ADEPD), Volledig gereviseerde versie 2019.

[28] Federatie Medisch Specialisten: Richtlijn, Preventie van valincidenten bij ouderen. In.; 2017.

[29] VeiligheidNL (2020). Valanalyse, Zo beoordeel je valrisico bij ouderen.

[30] World Health Organization (2008). Ageing & Life Course Unit: WHO global report on falls prevention in older age.

[31] Gobbens RJ, van Assen MA, Luijkx KG, Wijnen-Sponselee MT, Schols JM (2010). The Tilburg frailty indicator: psychometric properties. J Am Med Dir Assoc.

[32] Peters LL, Boter H, Buskens E, Slaets JP (2012). Measurement properties of the Groningen frailty Indicator in home-dwelling and institutionalized elderly people. J Am Med Dir Assoc.

[33] Hoogendijk EO, Van Der Horst HE, Deeg DJ, Frijters DH, Prins BA, Jansen AP, Nijpels G, Van Hout HP (2013). The identification of frail older adults in primary care: comparing the accuracy of five simple instruments. Age Ageing.

[34] Coll-Planas L, Kron M, Sander S, Rißmann U, Becker C, Nikolaus T (2006). Accidental falls among community-dwelling older adults. Z Gerontol Geriatr.

[35] Ersoy Y, MacWalter RS, Durmus B, Altay ZE, Baysal O (2009). Predictive effects of different clinical balance measures and the fear of falling on falls in postmenopausal women aged 50 years and over. Gerontology.

[36] Gerdhem P, Ringsberg KA, Åkesson K, Obrant KJ (2005). Clinical history and biologic age predicted falls better than objective functional tests. J Clin Epidemiol.

[37] Lindemann U, Lundin-Olsson L, Hauer K, Wengert M, Becker C, Pfeiffer K (2008). Maximum step length as a potential screening tool for falls in non-disabled older adults living in the community. Aging Clin Exp Res.

[38] Nitz J, Stock L, Khan A (2013). Health-related predictors of falls and fractures in women over 40. Osteoporos Int.

[39] Pluijm SM, Smit JH, Tromp E, Stel V, Deeg DJ, Bouter LM, Lips P (2006). A risk profile for identifying community-dwelling elderly with a high risk of recurrent falling: results of a 3-year prospective study. Osteoporos Int.

[40] Tiedemann A, Lord SR, Sherrington C (2010). The development and validation of a brief performance-based fall risk assessment tool for use in primary care. J Gerontol A Biomed Sci Med Sci.

[41] StataCorp (2017). Stata statistical software: release 15.

[42] Schoon Y (2013). Vallen, een systematische benadering bij het zoeken naar de oorzaak. Bijblijven.

[43] Seymour D (2018). One small step for older people with frailty, one giant leap for frailty care? An analysis of GP contract services data for routine frailty identification and frailty care through the GP contract 2017/2018.

[44] Stel VS, Smit JH, Pluijm SM, Lips P (2004). Consequences of falling in older men and women and risk factors for health service use and functional decline. Age Ageing.

[45] Ensrud KE, Ewing SK, Taylor BC, Fink HA, Stone KL, Cauley JA, Tracy JK, Hochberg MC, Rodondi N, Cawthon PM (2007). Frailty and risk of falls, fracture, and mortality in older women: the study of osteoporotic fractures. J Gerontol Ser A Biol Med Sci.

[46] Campbell AJ, Robertson MC (2006). Implementation of multifactorial interventions for fall and fracture prevention. Age Ageing.

[47] Wolf SL, Sattin RW, Kutner M, O'Grady M, Greenspan AI, Gregor RJ (2003). Intense tai chi exercise training and fall occurrences in older, transitionally frail adults: a randomized, controlled trial. J Am Geriatr Soc.

[48] Bunn F, Dickinson A, Barnett-Page E, Mcinnes E, Horton K (2008). A systematic review of older people’s perceptions of facilitators and barriers to participation in falls-prevention interventions. Ageing Soc.

[49] Loganathan A, Ng CJ, Tan MP, Low WY (2015). Barriers faced by healthcare professionals when managing falls in older people in Kuala Lumpur, Malaysia: a qualitative study. BMJ Open.

[50] Paul S. Falls: prevention and management. Geriatr Med. 2018:109–19.

[51] Chen S-F, Huang S-F, Lu L-T, Wang M-C, Liao J-Y, Guo J-L (2016). Patterns of perspectives on fall-prevention beliefs by community-dwelling older adults: a Q method investigation. BMC Geriatr.

[52] McMahon S, Talley KM, Wyman JF (2011). Older people’s perspectives on fall risk and fall prevention programs: a literature review. Int J Older People Nursing.

[53] Sherrington C, Michaleff ZA, Fairhall N, Paul SS, Tiedemann A, Whitney J, Cumming RG, Herbert RD, Close JC, Lord SR (2017). Exercise to prevent falls in older adults: an updated systematic review and meta-analysis. Br J Sports Med.

[54] Olij BF, Ophuis RH, Polinder S, Van Beeck EF, Burdorf A, Panneman MJ, Sterke CS (2018). Economic evaluations of falls prevention programs for older adults: a systematic review. J Am Geriatr Soc.

[55] Bij S, Biermans M, Khan N, Akkermans R, Peters H, Levelink H, Verheij R: De kwaliteit van de verslaglegging in medische dossiers: uitbreiding van de EPD-scan. Regio Nijmegen, eerste meting. 2013.

[56] Donker: NIVEL primary care database-sentinel practices. 2016.

