# Supplementary material for: Falls prevention at GP practices: a description of daily practice
Source: BMC Fam Pract. 2021 Sep 21;22:190. doi: 10.1186/s12875-021-01540-7 (PMC8454103; doi:10.1186/s12875-021-01540-7)
Supplement: Supplementary file 1 — Additional file 1. Selected ICPC-codes related to frailty and/or high fall risk [file 12875_2021_1540_MOESM1_ESM.docx]

# **Falls prevention at GP practices: A description of daily practice**

Wytske M.A. Meekes, Chantal J. Leemrijse, Yvette M. Weesie, Ien A.M. van de Goor, Gé A. Donker, Joke C. Korevaar

## **Additional file 1: Selected ICPC-codes related to frailty and/or high fall risk**

| **ICPC-Code** | **Description** | **ICPC-Code** | **Description** |
| --- | --- | --- | --- |
| **A** | **General and unspecified** | L74.01 | Fracture: phalanges hand/broken fingers |
| A05 | Feeling ill/ Overall decline/ Frailty | L74.02 | Fracture: phalanges foot/ broken toes |
| A06 | Fainting/syncope | L75 | Fracture: femur/ broken thigh |
| A49 | Other preventive operations | L75.01 | Fracture: collum femoris/ hip fracture |
| A49.01 | Elderly care |  |  |
| A49.02 | Polypharmacy | L76 | Fracture: other |
| A80 | Trauma/ injury NOS/ Falls | L76.03 | Fracture: clavicle/ broken collarbone |
| A81 | Multiple trauma/ injuries | L76.04 | Fracture: humerus/ broken upper arm |
| **F** | **Eye** | L76.05 | Fracture: rib |
| F84 | Macular degeneration | L76.06 | Fracture: spine |
| F94 | Blindness | L76.07 | Fracture: pelvis |
| **H** | **Ear** | L76.08 | Fracture: patella |
| H82 | Vertiginous syndrome | L77 | Sprain/strain of ankle |
| H82.01 | [Ménière’s disease/syndrome](https://en.wikipedia.org/wiki/M%C3%A9ni%C3%A8re%27s_disease) | L78 | Sprain/strain of knee |
| H82.02 | Labyrinthitis/ vestibular neuritis | L81 | Injury musculoskeletal NOS |
| H82.03 | Benign paroxysmal positional vertigo | L81.01 | Coup de fouet/ whiplash |
| **K** | **Circulatory** | L81.02 | Rib contusion |
| K77 | Heart failure | L84 | Back syndrome without radiating pain/ Osteoarthritis spine /spondylosis |
| K77.01 | Acute decompensated heart failure / cardiac asthma | L84.01 | Osteoarthritis/spondylosis |
| K77.02 | Heart failure/ Chronic decompensatio cordis | L89 | Osteoarthrosis of hip |
| K78 | Atrial fibrillation/flutter | L90 | Osteoarthrosis of knee |
| K79 | Paroxysmal tachycardia | L91 | Osteoarthrosis other |
| K79.01 | Supraventricular tachycardia | L95 | Osteoporosis |
| K88 | Postural hypotension | L95.01 | Osteopenia/ low bone density |
| K90 | Stroke/cerebrovascular accident | L95.02 | Osteoporosis |
| K90.01 | Subarachnoid haemorrhage | **N** | **Neurological** |
| K90.02 | Intracerebral hemorrhage/ cerebral bleeding | N17 | Vertigo/dizziness |
| K90.03 | Cerebral infarction | N17.01 | Vertigo |
| **L** | **Muscoloskeletal** | N17.02 | Light-headedness |
| L49.01 | Falls prevention/ fracture prevention | N86 | Multiple sclerosis |
| L72 | Fracture: radius/ ulna | N87 | Parkinsonism |
| L73 | Fracture: tibia/fibula | N87.01 | Parkinson’s disease |
| L74 | Fracture: hand/foot bone | N99 | Neurological disease, other |
