# Supplementary material for: Falls prevention at GP practices: a description of daily practice
Source: BMC Fam Pract. 2021 Sep 21;22:190. doi: 10.1186/s12875-021-01540-7 (PMC8454103; doi:10.1186/s12875-021-01540-7)
Supplement: Supplementary file 2 — Additional file 2. Questionnaire used to investigate GPs daily practice regarding falls prevention [file 12875_2021_1540_MOESM2_ESM.docx]

# Falls prevention at GP practices: A description of daily practice

Wytske M.A. Meekes, Chantal J. Leemrijse, Yvette M. Weesie, Ien A.M. van de Goor, Gé A. Donker, Joke C. Korevaar

**Additional file 2: Questionnaire used to investigate GPs daily practice regarding falls prevention**

1. Based on what did you diagnose the patient as being frail?

- Based on my clinical expertise
  - Based on an instrument that assesses patients’ frailty (e.g. Tilburg Frailty Indicator, Groninger Frailty Indicator)

2a. Did the patient fall during the past 12 months?

- Yes
- No
- Unkown

2b. Does the patient have a fear of falling?

- Yes
- No
- Unkown

3. Does the patient receive treatment regarding high fall risk?

- No 🡪 go to question 4
- Yes🡪 go to question 5

1. Why does this patient not receive fall preventive care?

- The patient has no high fall risk
- I believe the patient has a high fall risk, however the patient experiences this differently
- Both patient and I believe the patient has a high fall risk, however the patient experiences the treatment to intensive or too much of a hassle
- Both patient and I believe the patient has a high fall risk, however the treatment costs are too high
- Other, namely……………………………………………………………………………………………………………………

1. For which fall risk factors does the patient receive care?

- Limitation in mobility/ADL/physical activity
- Fear of falling
- Medication (polypharmacy, and/or use of benzodiazepines / sleeping medication)
- Impaired vision
- Dizziness
- Incontinence
- Wrong use of walking aid
- Footwear
- Depressive/Cognitive problems (e.g. forgetfulness)
- Cardiovascular factors
- Syncope
- Osteoporosis
- Alcohol use
- Other, namely……………………………………………………………………………………………………………………
- Other, namely……………………………………………………………………………………………………………………
- Other, namely……………………………………………………………………………………………………………………

1. Which health care providers offer this patient fall preventive care?

- Myself (GP)
- Practice nurse
- Home care/District nurse
- Occupational therapist
- Physiotherapist
- Exercise therapist
- Pharmacist
- Podiatrist
- Psychologist
- Geriatrician
- Cardiologist
- Optician
- Other, namely……………………………………………………………………………………………………………………
- Other, namely……………………………………………………………………………………………………………………
- Other, namely……………………………………………………………………………………………………………………
