# Supplementary material for: Falls prevention at GP practices: a description of daily practice
Source: BMC Fam Pract. 2021 Sep 21;22:190. doi: 10.1186/s12875-021-01540-7 (PMC8454103; doi:10.1186/s12875-021-01540-7)
Supplement: Supplementary file 3 — Additional file 3. Flowchart fall preventive care offered to frail older patients [file 12875_2021_1540_MOESM3_ESM.docx]

# Falls prevention at GP practices: A description of daily practice

Wytske M.A. Meekes, Chantal J. Leemrijse, Yvette M. Weesie, Ien A.M. van de Goor, Gé A. Donker, Joke C. Korevaar

**Additional file 3: Flowchart fall preventive care offered to frail older patients**


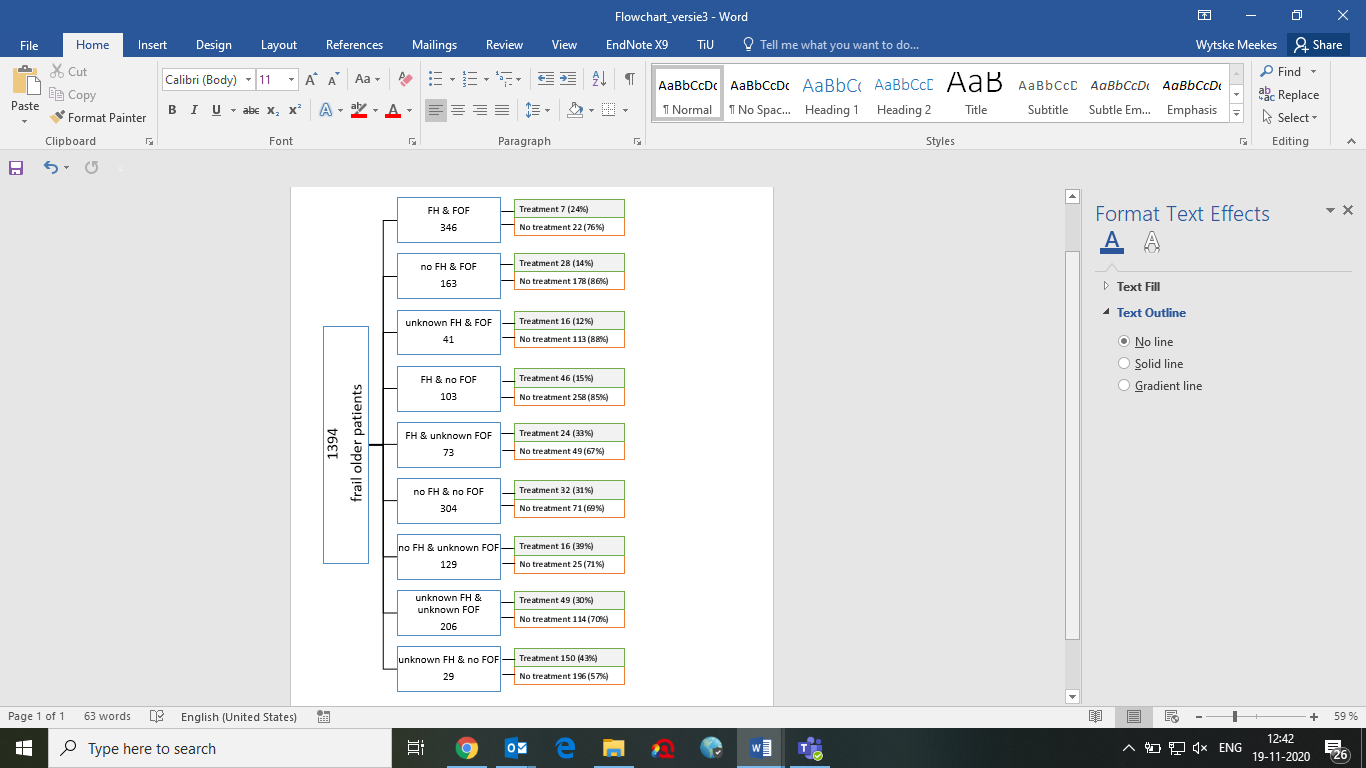


FH: Fall History FOF: Fear of Falling
